# A Comparative Analysis of Skin Cancer Detection Applications Using Histogram-Based Local Descriptors

**DOI:** 10.3390/diagnostics13193142

**Published:** 2023-10-06

**Authors:** Yildiz Aydin

**Affiliations:** Department of Computer Engineering, Erzincan Binali Yildirim University, Erzincan 24000, Turkey; yciltas@erzincan.edu.tr

**Keywords:** identification, skin cancer, histogram-based local descriptors, color features

## Abstract

Among the most serious types of cancer is skin cancer. Despite the risk of death, when caught early, the rate of survival is greater than 95%. This inspires researchers to explore methods that allow for the early detection of skin cancer that could save millions of lives. The ability to detect the early signs of skin cancer has become more urgent in light of the rising number of illnesses, the high death rate, and costly healthcare treatments. Given the gravity of these issues, experts have created a number of existing approaches for detecting skin cancer. Identifying skin cancer and whether it is benign or malignant involves detecting features of the lesions such as size, form, symmetry, color, etc. The aim of this study is to determine the most successful skin cancer detection methods by comparing the outcomes and effectiveness of the various applications that categorize benign and malignant forms of skin cancer. Descriptors such as the Local Binary Pattern (LBP), the Local Directional Number Pattern (LDN), the Pyramid of Histogram of Oriented Gradients (PHOG), the Local Directional Pattern (LDiP), and Monogenic Binary Coding (MBC) are used to extract the necessary features. Support vector machines (SVM) and XGBoost are used in the classification process. In addition, this study uses colored histogram-based features to classify the various characteristics obtained from the color images. In the experimental results, the applications implemented with the proposed color histogram-based features were observed to be more successful. Under the proposed method (the colored LDN feature obtained using the YCbCr color space with the XGBoost classifier), a 90% accuracy rate was achieved on Dataset 1, which was obtained from the Kaggle website. For the HAM10000 data set, an accuracy rate of 96.50% was achieved under a similar proposed method (the colored MBC feature obtained using the HSV color space with the XGBoost classifier).

## 1. Introduction

Lesions are defined as areas of the skin that are abnormal in comparison to other areas of the skin. Infections that occur in or on the skin are the fundamental and primary cause of lesions. Lesions can be classified into two categories: malignant (melanoma) and benign. Benign lesions are not as harmful since they develop slowly and do not spread. Physical identification of melanoma with the naked eye is not particularly effective since the characteristics of the lesion cannot be observed thoroughly and could lead to maltreatment or even death. Overall survival from skin cancer is directly proportionate to how early malignant lesions are detected. In order to enhance accuracy and effectiveness, reliable automated analysis is crucial. Dermoscopic imaging methods are created to accurately detect the location of skin lesions, and by reducing reflection, the visual images are improved. Nevertheless, there are a few challenges in the automated classification of skin lesions, such as artifacts, poor contrast, color of skin, veins, and comparable visuals of melanoma and nonmelanoma.

Malignant skin cancer poses a serious health risk to individuals across the spectrum. The stage of the cancer at the moment of diagnosis has a significant impact on the chance of survival. If skin cancer is discovered before it begins to spread, there is a high probability of survival; but if it spreads to other systems in the body, there is a much lower likelihood of successful surgical intervention and a much higher rate of mortality. For this reason, the key to delaying the progression of skin cancer and achieving effective treatments is early detection. Skilled dermatologists will often perform a sequence of procedures, starting with a visual examination of any questionable symptoms, followed by a microscopic examination (microscopic magnification of the lesions), and finally, a biopsy. Unfortunately, this lengthy process might cause the illness to progress to more advanced stages. Additionally, the exact diagnosis can be a somewhat subjective decision that is often based on the clinician’s background. However, if an automatic skin cancer recognition system is integrated into the diagnostic process by a hospital or dermatologist, there are a number of critical advantages. This process would typically involve skin-lesion images uploaded to the system by an assistant or dermatologist. The advantages of this system are a more efficient system that reduces human workload and, more critically, it increases the reliability of an accurate diagnosis.

A lot of effort has been carried out to create effective algorithms for machine imagery analysis so that melanoma can be identified in its preliminary phase and prevent some of the issues mentioned previously. Recently, machine learning and deep learning methods have been explored in many fields [1,2], including the health field. Although deep learning methods, which are frequently used in skin cancer detection applications, perform better than machine learning methods with high-dimensional data, they may perform less effectively than machine learning methods with low-dimensional data [3,4]. In this study, machine learning methods were used to measure accuracy with both low-dimensional data and high-dimensional data. The accuracy rate in machine learning methods is closely related to the features being analyzed. For this reason, many methods have been proposed recently that allow the features to be detected more clearly [5,6,7]. Typically, images used in classical feature extraction methods are initially color images, but they are first converted to grayscale images, and then the features of the image are extracted. One of the methods that allow the features to be detected more clearly is to use colored images instead of grayscale images in the feature extraction stage. This study suggests that a more accurate model of detecting skin cancer can be achieved by obtaining the features using a color histogram-based descriptor. This particular method has not been encountered in previous research and in the literature review. Many suggestions for obtaining colored features of the classical application process have been implemented in the keypoint-based feature extraction method [5,6]. The proposed method in this study to obtain color features was initially inspired by the research of A. Verma et al. [7]. The procedure used in their research for color SIFT extraction is applied for colored histogram-based feature extraction. This study seeks to identify accurate skin cancer detection applications by comparing the effectiveness of machine learning and deep learning methods.

Various methods can be applied to improve the performance of the classical machine learning methods. The following methods can be used to improve the performance of the classifier:

- **Data preprocessing:** Data preprocessing is one of the factors that significantly affect the performance of the model. It consists of some processing steps, such as normalizing data or removing unnecessary data.
- **Hyperparameter adjustment:** In order to optimize the model, hyperparameters such as the number of trees, maximum depth, and learning speed must be adjusted.
- **Feature selection:** Unnecessary features can be removed to increase the performance of the model, or various methods can be used to obtain stronger features.

In this study, the methods above were implemented to improve the classification performance and to improve the XGBoost classifier used in the proposed method. The biggest contribution was the use of colored histogram-based features, which have not been encountered in the literature previously. In the classical method, even if the images that are used to extract the histogram-based features are in color form, the data are first converted to a grayscale image in the preprocessing step, and then the feature is extracted. This can cause important information to be lost. The classical method can be detrimental, especially in cases where the classifier fails due to an unbalanced dataset. This study aims to develop and improve the classification process to obtain more robust features by extracting and combining features using all the color channels of the image instead of using grayscale images.

The findings show that the applied method is considerably more accurate than traditional methods while maintaining a sufficient level of usability. The following is a summary of the main findings of this study:

- The problem of skin cancer classification using classical machine-learning classification methods has been addressed by many researchers. However, it is generally classified as cancerous or non-cancerous. In this study, cancer types were also classified.
- The study proposes a new method based on combining histogram-based descriptors in different color spaces as a new and highly effective approach for classifying various types of cancer. Although key point-based features are frequently used in color images, the use of global features in color images has not been found in the literature. In addition, it has been observed that the accuracy rate when using color images increases considerably compared to the features obtained from grayscale images.
- Even though the dataset that used the proposed color histogram-based features was imbalanced, the classification was successful and did not suffer from the imbalance of the dataset.
- The success rates of color histogram-based descriptors are compared using different color spaces whose channels are not correlated.
- In comparison to existing methods, the applied method achieved greater efficiency.
- The proposed method was successful in both low-dimensional and high-dimensional data and was particularly effective in classifying low-dimensional data.
- In addition to the low-dimensional dataset [8], the proposed method was tested using the standard dataset HAM10000 [9], which is frequently used in the recent literature. The effectiveness of the proposed method was compared with other methods that have been recently proposed.

In the research, support vector machines (SVM) and XGBoost methods were used as classifiers, and the Local Binary Patterns (LBP), Local Directional Number Pattern (LDN), Pyramid of Histogram of Oriented Gradients (PHOG), Local Directional Pattern (LDiP) and Monogenic Binary Coding (MBC) methods were used as descriptors. In addition, a convolutional neural network and the Xception methods, which are deep learning methods, were also used for identifying skin cancer. This study will review the relevant literature in Section 2 and discuss the methods in depth in Section 3. The experimental data are described in Section 4, and the conclusions are detailed in Section 5.

## 2. Literature Review

Early detection of cancer is crucial in preventing the spread of cancer to other regions of the body and in the success of potential treatment. For this reason, automated methods of detecting skin cancer are a highly studied topic.

A study by Bakheet and Al-Hamadi describes an automated detection approach using Gabor-based features to analyze dermoscopic images [10]. The approach was found to have performed well at identifying the various types of skin cancers. The Google Inception v4 CNN’s structure was developed and confirmed for use in a skin cancer detection system by Haenssle et al. [11]. They combined the images with clinical information in two phases. Dermoscopic images were used in the initial phase, while both dermoscopic images and medical evidence were employed in the second phase. The main output of this method is a categorical diagnostic algorithm that operates using dermoscopic images. In another study, Hasan et al. [12] preprocessed the images they analyzed with a convolutional neural network classifier and achieved an 87.6% success rate. Esteva et al. [13] also examined around 120 thousand photos that were used for training in CNN architecture. In the study, a region of interest (ROI) and a transfer learning technique were employed to detect skin cancer, and they were able to attain dermatologist-level diagnostic accuracy [14]. Similarly, Muruguan et al. [15] proposed a four-stage skin cancer detection method: preprocessing, segmentation, feature extraction, and classification. The method using SVM and K-nearest neighbor classifiers performed very efficiently in the classification process. Background information is also included with the skin lesion images and is used in the classification process. The success rate in skin cancer classification may vary depending on the segmentation of the skin lesions and the background [16]. Venugopal et al. [17] proposed a CNN-based method to obtain border information by detecting the location of skin lesions. In their proposed method, CNN was used for threshold estimation, and their segmentation process was very successful.

While some researchers in the literature analyzed a dataset that they created, other researchers used standard datasets. Some of the popular datasets are ISIC, HAM10000, PH2, MED-NODE, and the DermIS datasets [18]. Sharafudeen and Chandra [19] presented a new method using the features they obtained with data from the patients and with data from EfficientNets. In the method they proposed, they obtained an accuracy rate of 94.13% in the ISIC 2018 dataset and 91.93% in the ISIC 2019 dataset. Others, such as Kousis et al., proposed a method using the HAM10000 database in 11 CNN architectures in order to classify seven skin lesions [20]. DenseNet169 was found to be the most successful method, with an accuracy rate of 92.25%. A CNN-based model was proposed by Özbay and Altunbey Özbay [21] to classify skin lesions as either benign or malignant using the HAM10000 dataset. Since there was an imbalance between the image numbers of the classes in the HAM10000 dataset, they divided the dataset into two groups, either malignant or benign, and achieved a success rate of 99.69%. Özbay and Altunbey Özbay also developed a skin cancer detection application with the Optimized CNN method using the ISIC-2019 and Asian-dermoscopy datasets. The particle swarm optimization method was used for optimization, and the skin cancer classification application they suggested achieved a 99.33% rate of accuracy. Furthermore, Keerthana et al. [22] proposed a new skin cancer classification method using a hybrid of the CNN and SVM methods together. Their proposed method uses CNN for feature extraction and SVM for classification, and the model maintained a success rate of 88.02% with the ISBI 2016 dataset.

## 3. Proposed Method

**Figure 1:**
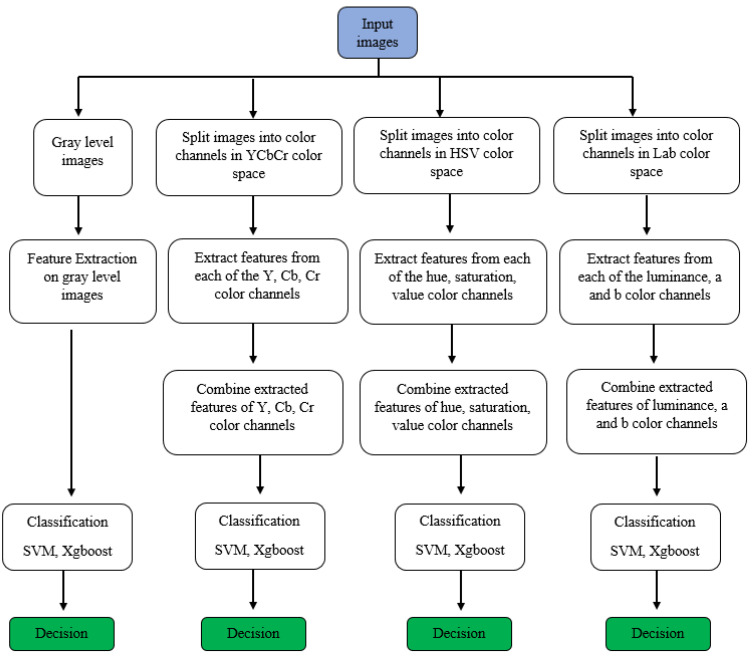
The research progression of the recommended approach.

The objective of this study is to use methods of histogram-based local descriptors to detect skin cancer. In this study, the LBP, LDN, PHOG, LDiP, and MBC local descriptors were used in the feature extraction step, and the SVM and XGBoost methods were used in the classification step. In addition, when used as a feature in the application process, hybrid features were obtained by combining the features with the highest accuracy and f1 values. In addition, in this study, colored histogram-based features obtained using color images instead of grayscale images were used in the process of identifying skin cancer. Historically, in the feature extraction process, regardless of whether the images are colored or not, they are first converted to grayscale, and then the features are extracted. However, more recently, colored features have been proposed as more effective in extracting detailed features. While extracting colored features, the HSV color space is used in some studies [23], LAB color space is used in other studies [24], and YCbCr color space is used in others as well [25]. In this study, HSV, LAB, and YCbCr color spaces were used for more comprehensive research and comparison. All channels of each color space are used to obtain the colored feature in that color space. Figure 1 illustrates the research progression of the recommended approach. Specifics on the methods used in this article are detailed below.

### 3.1. Histogram-Based Local Descriptors

Histogram-based regional classifiers generate local statistical information at critical places and use a summary form to characterize the particulars of an area. The central pixel of a local area is represented as a decimal value in histogram-based local descriptors depending on its own values with respect to the surrounding pixels. Regional variation programming is a common technique for encoding the features of the patterns in a regional patch independently of the input image. Common local variety coding has five phases for every local patch in a specific neighborhood: linear filtration, quantification, binarization, coding, and binary-to-decimal conversion [26].

### 3.2. Hybrid Histogram-Based Local Descriptors

Combining features ([27,28]) is very popular in image processing applications. Although the use of colored features has increased considerably in recent years, it has generally been used in keypoint-based features [25,29]. However, in this study, histogram-based features are used for the color images. In Equations (1) and (2), the formula for obtaining the hybrid feature and colored features, respectively, is given by showing the LBP and MBC features as examples.

(1) Hybrid Feature=LBP+MBC

(2) $${\mathrm{Color}\;\mathrm{LBP}}_{\mathrm{YCbCr}}={\mathrm{LBP}}_{Y}+{\mathrm{LBP}}_{\mathrm{Cb}}+{\mathrm{LBP}}_{\mathrm{Cr}}$$

In Equation (2), ${\mathrm{LBP}}_{Y}$ is the LBP feature extracted from the Y channel, ${\mathrm{LBP}}_{\mathrm{Cb}}$ is the LBP feature extracted from the Cb channel and ${\mathrm{LBP}}_{\mathrm{Cr}}$ is the LBP feature extracted from the Cr channel. Color features are obtained in a similar way using other color channels.

**Figure 2:**
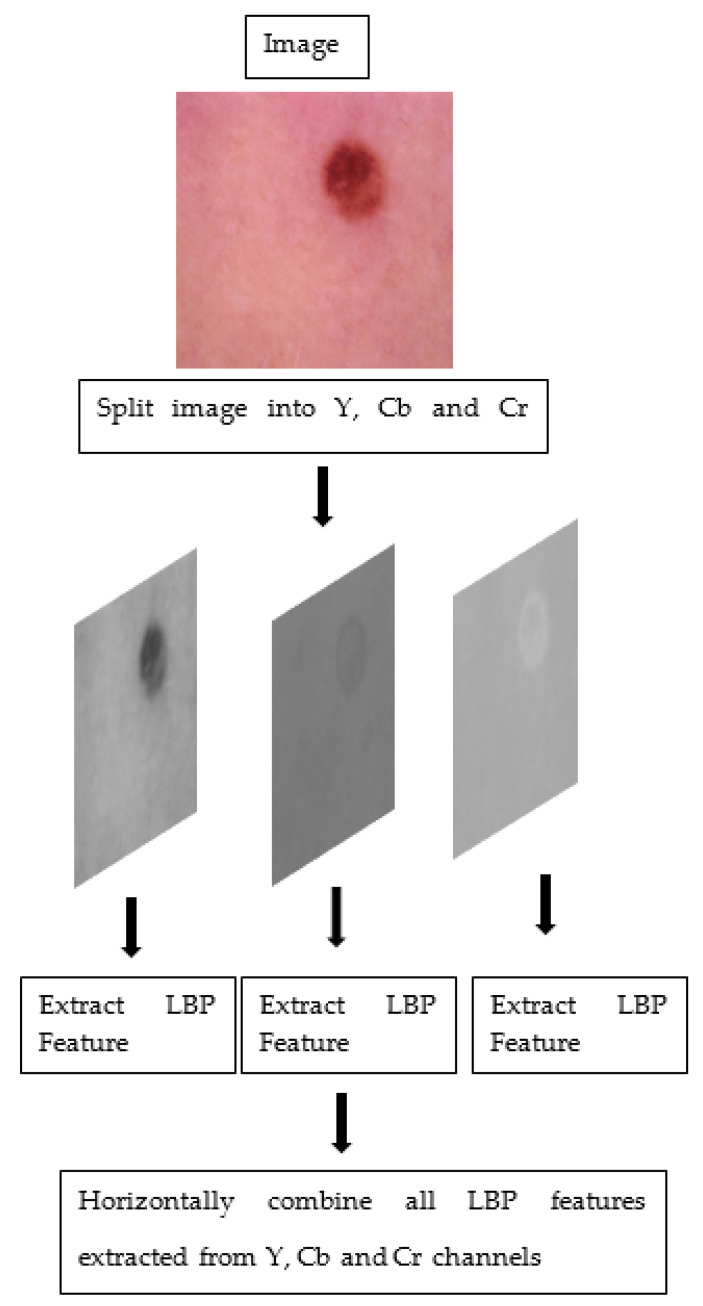
The steps taken to obtain colored features.

The steps taken to obtain colored features on a sample image are given in Figure 2.

### 3.3. Support Vector Machines

The objective of the SVM algorithm is to establish the optimal vector or set point that could also divide the n-dimensional area into categories, allowing us to quickly classify datasets. There are two types of support vector machines since data are linearly separable and non-separable. In linear support vector machines, the hyperplane that maximizes the distance between the categories is calculated. A hyperplane is the name given to the boundaries of this optimal path. SVM selects the extreme vectors that assist in the creation of the hyperplane. In the optimization process used to find this hyperplane, the formula given in Equation (3) is used.

(3) $$w\cdot x_{i}+b\geq +1\;IF\;y_{i}=+1\;w\cdot x_{i}+b\leq -1\;IF\;y_{i}=-1$$

In Equation (3), w is the weight vector, x is the input vector, b is the bias, and y is the class label. The optimum hyperplane is determined using the two necessary hyperplanes to form the boundaries. The points on these hyperplanes are called support vector machines. In order to maximize the distance between support vector machines, the $\left|\left|w\right|\right|$ expression must be minimized. In order to achieve this, the optimization problem given in Equation (4) must be solved.

(4) $$\min\left[\frac{1}{2}{\left|\left|w\right|\right|}^{2}\right]$$

This optimization problem can be solved using Lagrange multipliers. Using the result, the decision function is calculated using the formula shown in Equation (5).

(5) $$f\left(x\right)=sign\left(\sum_{i=1}^{k}\;\lambda_{i}y_{i}\left(x\cdot x_{i}\right)+b\right)$$

Here, $\lambda_{i}$ represents the Lagrange coefficients. In sample space problems that cannot be linearly separated, SVM uses kernel functions to move the sample space to another space where it can be linearly separated [30,31]. These functions are the Linear Kernel function, the Polynomial Kernel function, the Radial Basis Function (RBF), the Kernel function, and the Sigmoid Kernel function.

### 3.4. Extreme Gradient Boosting (XGBoost)

The Extreme Gradient Boosting algorithm was proposed by Chen and Guestrin in 2016 [32]. The gradient-boosted decision tree (GBDT) algorithm was used to construct the XGBoost model in order to maximize speed and efficiency efficiency [33,34,35,36]. The benefit of XGBoost over GBDT is that it enables linear classifications and conducts Taylor expansion by adding the partial derivatives to improve the accuracy of the findings. 

The objective function is optimized using the XGBoost algorithm using an additional training schedule. This implies the outcome of the preceding step is taken into consideration during the optimization process. The objective of the system may be written as shown in Equation (6) [33].

(6) $$obj^{t}=\sum_{i}l\left(y_{pred,}^{\left(t\right)}y_{truth}\right)+\sum_{k}\Omega \left(f_{k}\right),$$

In Equation (6), l expresses the loss term in step t, and Ω is the regularization phrase of the model and is calculated using the formula shown in Equation (7). obj  is the result of the error function. A more meaningful decision tree is formed when the obj  value becomes smaller.

(7) $$\Omega \;\left(f_{k}\right)=\gamma T+\frac{1}{2}\lambda {\left|\left|w\right|\right|}^{2}$$

In Equation (7), T expresses the number of leaf nodes, w indicates the rating of the leaves, and  γ and λ express the parameter with respect to T and w.

## 4. Experimental Results and Discussion

This section presents and discusses the research findings regarding the effectiveness of the proposed skin cancer detection system. A library [26] was created within the Matlab R2022a program for feature extraction. Training and testing processes were conducted with the feature histograms, which were obtained using the default parameters for extracting features from the library. The size of the extracted feature histogram varies depending on the feature used. The size of the feature histogram obtained from each of the color channels in the LAB, HSV, and YCbCr color space is 256, 56, 168, 56, and 3072 for the LBP, LDN, PHOG, LDiP, and MBC features, respectively. In the classification step, the Sklearn library was used for the SVM and XBboost classifiers. The default parameters defined in this library are used, and no parameter selection was made. Matlab R2022a was used for feature extraction, and the Python scripting language was used for all other operations. The experiments were performed on a computer with an i7 processor and 16 GB of RAM.

The dataset, evaluation metrics, and the results of the experiments conducted within the scope of this study are detailed in the following subsections. 

### 4.1. Dataset

**Figure 3:**
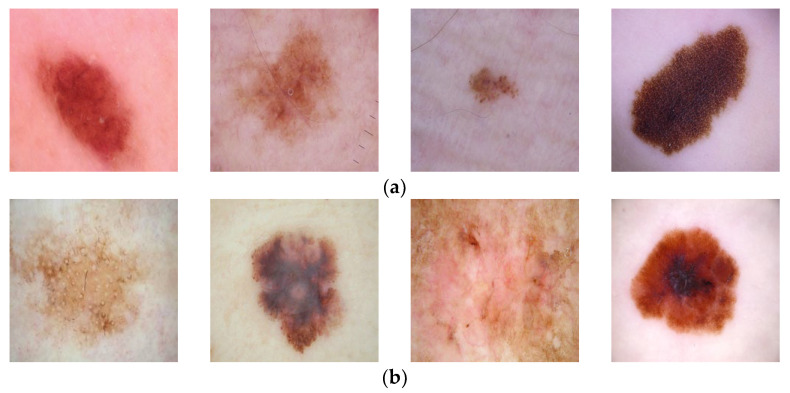
Examples of lesions from Dataset 1 [8]. (**a**) benign, (**b**) malignant.

In this study, two datasets are used. The first dataset is taken from the open-access website Kaggle [8] and is named Dataset 1 in this article. In this dataset, skin cancer types are classified as benign and malignant. In the training dataset, there are a total of 2637 images, of which 1440 images were categorized as benign, and 1197 images were categorized as malignant. In the test dataset, 360 images were classified as benign, and 300 images were classified as malignant. The resolution of these images is 224 × 224. Examples of lesion images from the dataset may be seen in Figure 3. The second dataset used is the HAM10000 dataset [9]. From this dataset, there are a total of 10015 images that are categorized into seven classes. From all these images, 327 images are classified as actinic keratosis, 514 as basal cell carcinoma, 1099 as benign keratosis, 115 as dermatofibroma, 6705 as melanocytic nevi, 142 as melanoma, and 1113 images are classified as vascular. For the experiments carried out within the scope of this study, the size of the images in the HAM10000 dataset is 28 × 28. Since there are not many studies on the first dataset in the literature, the HAM10000 dataset, which is frequently used in the literature, was also used, and the proposed method was compared with these more recent studies. The HAM10000 dataset with seven lesion categories is an imbalanced dataset. Some categories have quite a lot of images, while other categories have very few images. This may cause the machine-learning process to break down [37]. Machine learning methods tend to focus on categories that contain a high number of images and ignore the categories with a low number of images. There are various approaches and solutions to avoid this negative effect, such as the weighting method, ensemble methods, and deep learning methods. The process of oversampling is the one solution to the imbalance problem. As the data increases, the distribution of the samples into the various categories becomes more balanced. In this study, oversampling was performed using the SMOTE method [38] with the HAM10000 dataset.

### 4.2. Evaluation Parameters

The findings are quantitatively evaluated in relation to four commonly used quality parameters to measure the program’s performance: accuracy, precision, recall, and the F1 value.

Accuracy (AC) is the likelihood that the test data will provide the right conclusion. It is calculated using the formula given in Equation (8).

(8) $$Ac=\frac{TP+TN}{TP+TN+FP+FN}\times 100\%$$

The capacity to correctly detect cases of melanoma is referred to as recall (also known as the true positive rate, as seen in Equation (9)). Precision, on the other hand, gives the percentage of how many of the samples predicted as melanoma actually have melanoma (Equation (10)). The F1 value, whose formula is given in Equation (11), is a measurement metric obtained from integrating together the precision and recall values.

(9) $$\;\;\;Recall\;\left(r_{c}\right)=\frac{TP}{TP+FN}$$

(10) $$\mathrm{Precision}\;\left(p_{r}\right)=\frac{TP}{TP+FP}$$

(11) $$\;\;\;F1\;Score=2\times \frac{Recall\;*\;Precision}{Recall+Precision}$$

In the equations, true positive (*TP*) and true negative (*TN*) stand for the number of accurate predictions when the class value is true or false, respectively. False negative (*FN*) and false positive (*FP*) stands for the number of inaccurate predictions whenever the class value is true or false, respectively.

### 4.3. Results of Methods on Grayscale Images

In classical feature extraction methods, even when images in the dataset are colored, the images are first converted to grayscale to extract the features. For this reason, in this section, the experimental results of the methods that use the classical feature extraction process are provided. Within the scope of this study, a skin cancer detection system was developed using histogram-based descriptors such as the Local Binary Pattern (LBP), Local Directional Number Pattern (LDN), Pyramid of Histogram of Oriented Gradients (PHOG), Local Directional Pattern (LDiP), Monogenic Binary Coding (MBC) together with the SVM and XGboost classifiers. In the literature, it has been recently observed that more successful results have increased with the use of hybrid features [27,28]. For this reason, the features used in skin cancer detection applications that have high success rates were combined. The results obtained when using the histogram-based features on Dataset 1 are given in Table 1.

The results obtained by performing the classical feature extraction using the HAM10000 dataset are shown in Table 2.

**Table 1:** Results of the skin cancer detection applications according to the histogram-based local descriptors on Dataset 1.

Classifier	Features	Precision	Recall	F1-Score	Accuracy
SVM	LBP	0.76	0.65	0.70	0.75
	LDN	0.76	0.66	0.71	0.75
	PHOG	0.71	0.65	0.68	0.72
	LDiP	0.77	0.64	0.70	0.75
	MBC	0.74	0.72	0.73	0.76
	LBP + MBC	0.77	0.76	0.77	0.79
	LBP + PHOG	0.76	0.64	0.70	0,75
XGBoost	LBP	0.79	0.79	0.79	0.81
	LDN	0.71	0.75	0.73	0.75
	PHOG	0.80	0.72	0.76	0.79
	LDiP	0.75	0.70	0.72	0.75
	MBC	0.74	0.79	0.77	0.78
	LBP + MBC	0.78	**0.81**	0.80	0.81
	LBP + PHOG	**0.83**	0.80	**0.82**	**0.83**

**Figure 4:**
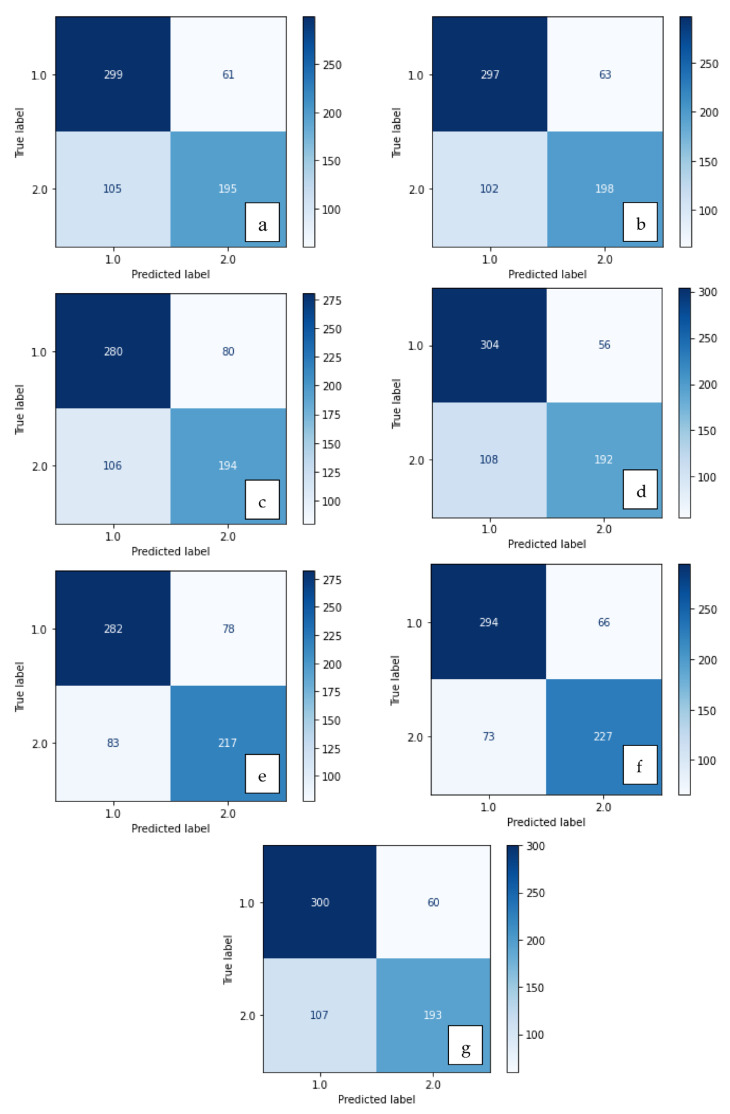
Confusion matrix of methods developed with SVM: (**a**) LBP, (**b**) LDN, (**c**) PHOG, (**d**) LDiP, (**e**) MBC, (**f**) LBP + MBC, (**g**) LBP + PHOG.

**Figure 5:**
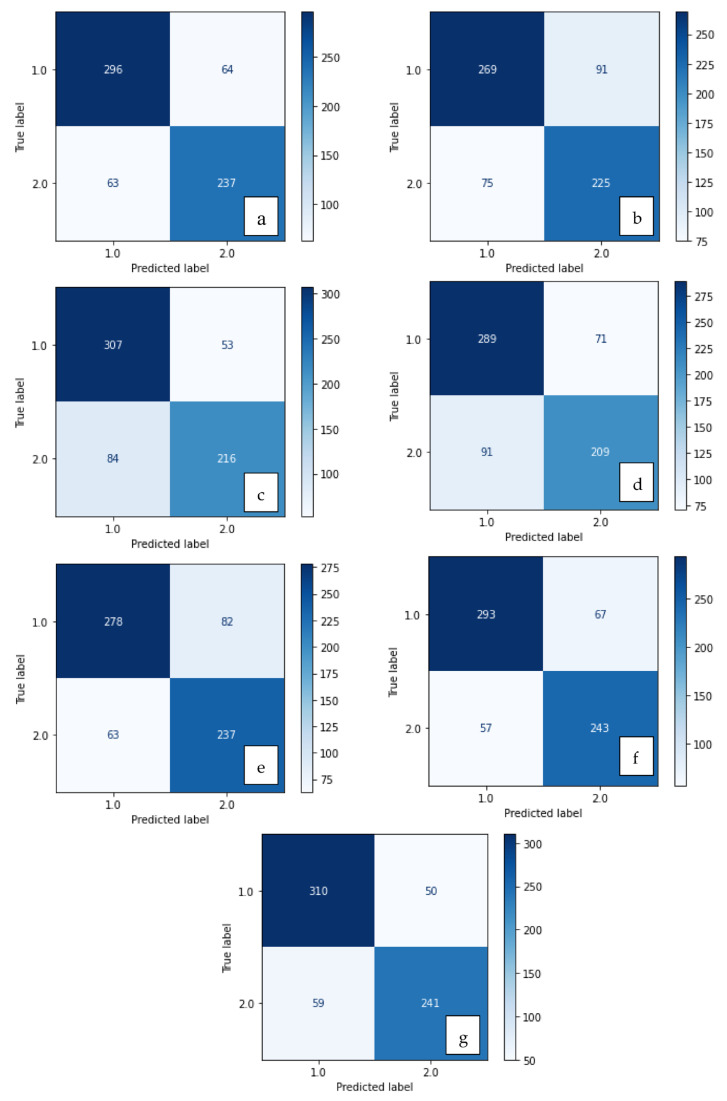
Confusion matrix of methods developed with XGBoost: (**a**) LBP, (**b**) LDN, (**c**) PHOG, (**d**) LDiP, (**e**) MBC, (**f**) LBP + MBC, (**g**) LBP + PHOG.

The confusion matrices of the methods are shown in Figure 4 and Figure 5, and their results are given in Table 1. As seen in Figure 4 and Figure 5 and Table 1, more accurate results are obtained when the features are used together rather than separately. Bold: the highest values.

### 4.4. Results of the Methods for Color Images

More recently, keypoint-based color features have been used frequently in the literature. It has been observed that the accuracy rates are higher than the features obtained from grayscale images. However, the use of colored features in a histogram-based analysis is not prevalent. In order to obtain the color feature, some studies have used the LAB color space [24], other studies have used the HSV color space [23], and others have also used the YCbCr color spaces [25]. This study uses these three color spaces to obtain a histogram-based color feature. The findings for Dataset 1 are shown in Table 3, Table 4 and Table 5.

Looking at the results given in Table 3, Table 4 and Table 5, it is observed that the most successful method depends on the color space used. In Table 3 and Table 4, the most successful method used the LAB and HSV color spaces and is the colored LBP feature and XGboost classifier. Table 5 shows the results of the YCbCr color space, and the most successful method is the colored LDN feature with the XGboost classifier. According to all the results shown in the tables, the method with the lowest success rate is with the colored PHOG feature obtained using the HSV color space together with the SVM classifier.

To extract histogram-based features, images are first converted to grayscale images, and then the features are extracted. Instead of using grayscale images, Table 5 shows that features from all channels of the YCbCr color space are extracted and combined. In this way, colored histogram-based features, which are not detailed in the literature, were used, and a stronger feature was obtained.

**Figure 6:**
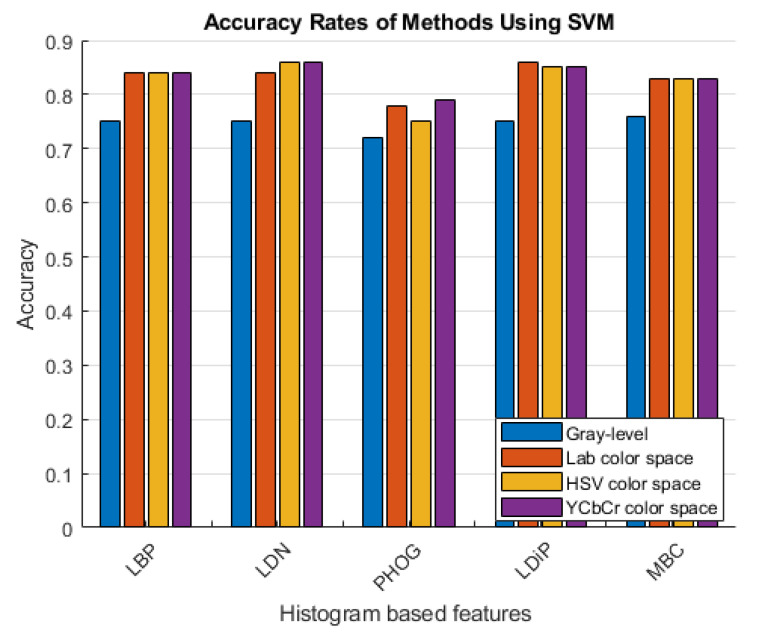
Accuracy rates of the methods developed with the SVM classifier.

**Figure 7:**
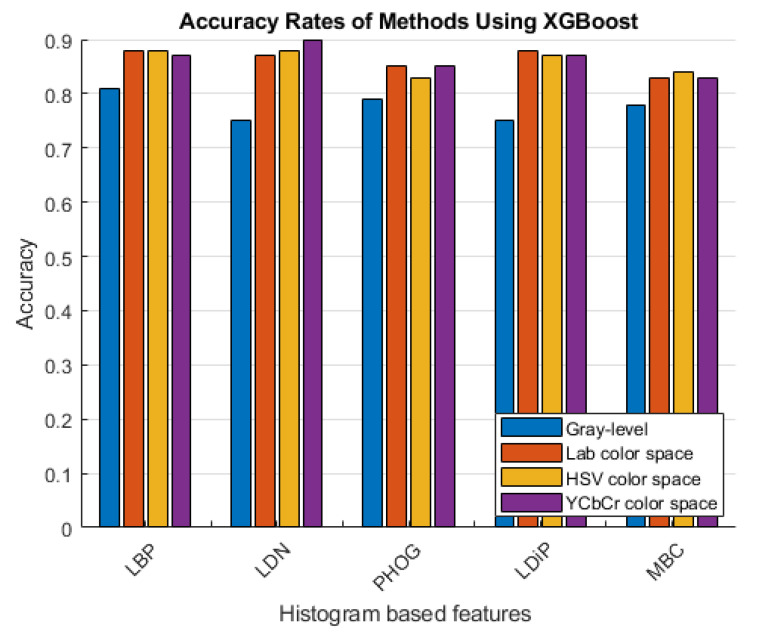
Accuracy rates of the methods developed with XGBoost.

The accuracy rates of the methods that use the histogram-based features obtained using the grayscale image and the colored histogram-based features obtained using the LAB, HSV, and YCbCr color channels are given in Figure 6 and Figure 7.

As can be seen in Figure 6 and Figure 7, more accurate results were obtained in the experiments performed with the colored features. These features were obtained using color spaces with no correlation between the color channels instead of using the classical feature extraction method.

The results of the experiments that used the color histogram-based descriptors on the HAM10000 dataset are shown in Table 6. According to the results of the HAM10000 dataset in Table 6, the most successful method is the colored MBC feature obtained using the HSV color space with the XGboost classifier. The method with the lowest accuracy rate is the method that uses the colored LDN feature obtained using the LAB color space together with the SVM classifier.

When using colored features, three color spaces were used separately, and the performance of histogram-based colored features was compared for each color space. If we look at the experimental results in Table 3, Table 4, Table 5 and Table 6, colored features in all three color spaces were more successful than the experiments carried out using grayscale images. Evaluating the results obtained with these three color spaces individually, it cannot be said that any specific color space obtained more successful results than another. The reasons are not only because the results are very close but also because the results obtained using the HSV color space were more successful with some features, while the results obtained using the YCbCr color space or Lab color space were more successful with other features.

In addition, to show that the applications are statistically significant, a two-tailed Welch’s t-test, often used in the literature [39,40], was applied. The application was developed using color histogram-based features, and it obtained successful results in both datasets. The labels predicted using the two classifiers are given as input, and the parameter was set to α = 0.05 (Table 7).

As seen in Table 7, There is no statistically significant difference between the two groups. The prediction labels obtained from the SVM classifier were subjected to a two-tailed Welch’s *t*-test with the prediction labels obtained from the SVM classifier, and the p-value was 1. Because we subjected the same results to this exact test. When we subjected the prediction results of the SVM classifier and the XGBoost classifier to a two-tailed Welch’s *t*-test, the *p* value was close to 1. This shows that the classifiers make errors on similar examples, so the results are consistent. The reason for unsuccessful prediction in the examples where errors are made may be due to various reasons, such as the complexity of the image or the feature not being able to describe this image at the desired level.

### 4.5. Comparative Results

The results of the proposed method using Dataset 1 and the Ham10000 datasets are given in Table 8 and Table 9, respectively. The CNN and Xception methods specified in Table 8 were conducted within the parameters of this study. The results for the proposed method are shown in Table 9, while the other results used for comparison are taken from T.M. Alam et al. [41], except for the results taken from Yang et al. [42].

The use of deep learning methods has become widespread in cancer detection systems, especially for large datasets. This area of research has been studied intensively in recent years [57,58,59]. Some of the leading methods are the MLP-Mixer method or the vision transformer model. In the experimental results obtained from the Ham10000 dataset in Table 8, the proposed method was observed to be more successful than the study that uses the vision transformer model [42]

As seen in Table 8 and Table 9, more accurate results were obtained with the proposed method when compared to the other previous methods.

## 5. Conclusions

This study conducted an analysis of applications used to identify skin cancer using histogram-based local descriptors. Various algorithms have been proposed in the literature to obtain stronger features. One of these algorithms is to analyze the features in their colored form. Many features such as SIFT, SURF, KAZE, ORB, HOG, and PHOG use grayscale images. For this reason, the use of color SIFT, color SURF, and many similar features have been proposed in the recent literature [23,60]. However, the specific use of colored histogram-based features has not been found in the literature. This study proposes the use of colored histogram-based features to solve some of the challenges with identifying skin cancer. As observed in the proposed method, more accurate results were obtained with colored histogram-based features, and it shows that it can also be used in solving different problems. According to the findings of the study, the suggested method works more effectively than traditional methods used to identify skin cancer. Even still, the quality of the image inputs has a strong impact on accuracy. In other words, poor-quality images might lead to significant errors in how accuracy rates are determined.

However, this study has some limitations. Although the success of the application increased with the use of colored features, the number of data required for processing also increased, and therefore, more resources were needed. In future studies, it is suggested that an effective skin cancer recognition application could be obtained by taking this negative effect into consideration. In a subsequent study, we intend to refine the proposed system to take other categories into account. Particularly, not using oversampling methods on imbalanced datasets when classifying and identifying skin diseases might carry some significant future advantages.

## Figures and Tables

**Table 2:** Results of the skin cancer detection applications according to the histogram-based local descriptors on HAM10000.

Classifier	Features	F1-Score	Accuracy
SVM	LBP	71.55	72.24
	LDN	3.52	14.17
	PHOG	3.52	14.17
	LDiP	74.60	75.09
	MBC	95.85	95.85
XGBoost	LBP	94.79	94.78
	LDN	3.52	14.17
	PHOG	3.52	14.17
	LDiP	93.85	93.85
	MBC	**96.09**	**96.00**

Looking at the results in Table 2, it is observed that the most successful method is the MBC feature and XGBoost classifier. In addition, since the classical feature extraction techniques were applied, the training was not successful because the dataset was unbalanced for the applications that use the LDN and PHOG features. Bold: the highest values.

**Table 3:** Results of the skin cancer detection applications according to the histogram-based local descriptors using the LAB color space on Dataset 1. Bold: the highest values.

Classifier	Features	Precision	Recall	F1-Score	Accuracy
SVM	LBP	0.79	0.88	0.83	0.84
	LDN	0.79	0.88	0.83	0.84
	PHOG	0.76	0.74	0.75	0.78
	LDiP	0.88	0.79	0.83	0.86
	MBC	0.78	0.85	0.82	0.83
XGBoost	LBP	0.85	**0.89**	**0.87**	**0.88**
	LDN	0.86	0.86	0.86	0.87
	PHOG	0.84	0.82	0.83	0.85
	LDiP	**0.88**	0.86	0.86	**0.88**
	MBC	0.80	0.84	0.82	0.83

**Table 4:** Results of the skin cancer detection applications according to the histogram-based local descriptors using HSV on Dataset 1. Bold: the highest values.

Classifier	Features	Precision	Recall	F1-Score	Accuracy
SVM	LBP	0.85	0.80	0.82	0.84
	LDN	0.83	0.86	0.85	0.86
	PHOG	0.71	0.76	0.73	0.75
	LDiP	0.82	0.87	0.84	0.85
	MBC	0.81	0.83	0.82	0.83
XGBoost	LBP	**0.86**	**0.89**	**0.87**	**0.88**
	LDN	0.86	0.87	0.87	0.88
	PHOG	0.80	0.85	0.82	0.83
	LDiP	0.85	0.87	0.86	0.87
	MBC	0.82	0.84	0.83	0.84

**Table 5:** Results of the skin cancer detection applications according to the histogram-based local descriptors using YCbCr on Dataset 1. Bold: the highest values.

Classifier	Features	Precision	Recall	F1-Score	Accuracy
SVM	LBP	0.86	0.78	0.81	0.84
	LDN	0.85	0.85	0.85	0.86
	PHOG	0.77	0.78	0.78	0.79
	LDiP	0.87	0.80	0.83	0.85
	MBC	0.78	0.88	0.83	0.83
XGBoost	LBP	0.86	0.86	0.86	0.87
	LDN	**0.89**	**0.89**	**0.89**	**0.90**
	PHOG	0.84	0.82	0.83	0.85
	LDiP	0.86	0.85	0.86	0.87
	MBC	0.80	0.84	0.82	0.83

**Table 6:** Results of the skin cancer detection applications according to the colored histogram-based local descriptors using HSV, LAB, and YCbCr color spaces with the HAM10000 dataset. Bold: the highest values.

Classifier	Features	F1-Score (HSV)	Accuracy (HSV)	F1-Score (LAB)	Accuracy (LAB)	F1-Score (YCbCr)	Accuracy (YCbCr)
SVM	LBP	88.91	89.28	86.57	86.91	87.87	88.15
	LDN	76.99	77.37	71.50	72.17	70.73	71.33
	PHOG	87.35	87.53	80.49	80.97	81.97	82.41
	LDiP	90.02	90.13	87.96	88.14	85.75	85.90
	MBC	96.31	96.45	96.35	**96.48**	**96.31**	**96.44**
XGBoost	LBP	95.82	95.87	95.85	95.83	96.07	96.05
	LDN	95.41	95.38	95.54	95.55	95.60	95.59
	PHOG	95.27	95.21	95.13	95.11	95.72	95.69
	LDiP	95.29	95.28	95.89	95.88	95.86	95.85
	MBC	**96.48**	**96.50**	**96.50**	96.42	96.12	96.04

**Table 7:** Results of the two-tailed Welch’s t-test.

Dataset	Features	Classifier	p-Value (SVM)	t-Value (XGBoost)
Dataset 1	LDN_YCbCr_	SVM	1	0
		XGBoost	0.868	−0.165
HAM10000	MBC_HSV_	SVM	1	0
		XGBoost	0.215	−1.23

**Table 8:** Results of the skin cancer detection applications using the same dataset (Dataset 1). Bold: the highest value.

Method	Accuracy Rates (%)
CNN	80.00
Xception	80.00
Kaya and Akgül [43]	83.00
Agarwal and Singh [44]	86.57
Soylu and Demir [45]	89.89
**Proposed method (colored LDN_YCbCr_ and XGBoost)**	**90.00**

**Table 9:** Results of the skin cancer detection applications using the HAM10000 dataset. Bold: the highest value.

Method	Accuracy Rates (%)
Fraiwan and Faouri [46]	82.9
Hoang et al. [47]	86.3
Popescu et al. [48]	86.7
Srinivasu et al. [49]	90.7
Khan et al. [50]	86.5
Huang et al. [51]	85.8
Khan et al. [52]	90.6
Thurnhofer-Hemsi et al. [53]	87.7
Xing et al. [54]	85.6
Chaturvedi et al. [55]	83.1
Ameri [56]	84.0
Alam et al. [41]	91.0
Yang et al. [42]	94.1
**Proposed method (colored MBC_HSV_ and XGBoost)**	**96.50**

## Data Availability

Data available on request from the authors.
